# Plastic rearrangement of basal forebrain parvalbumin-immunoreactive neurons in the kainite model of epilepsy

**DOI:** 10.3934/Neuroscience.2023023

**Published:** 2023-11-02

**Authors:** Ruben Carvalho, Alisa N. Lukoyanova, João Casalta-Lopes, Nikolay V. Lukoyanov, Joana Isabel Soares

**Affiliations:** 1 Master in Neurobiology, Faculty of Medicine, University of Porto, Porto, Portugal; 2 Neuronal Networks Group, Instituto de Investigação e Inovação em Saúde (i3S), University of Porto, Porto, Portugal; 3 Department of Biomedicine, Faculty of Medicine, University of Porto, Porto, Portugal; 4 Department of Basic Sciences, Polytechnic Institute of Coimbra, Coimbra Health School, Coimbra, Portugal; 5 Life and Health Sciences Research Institute / School of Medicine - University of Minho, Braga, Portugal; 6 Department of Radiotherapy, University Hospital Center of São João, Porto, Portugal

**Keywords:** temporal lobe epilepsy, kainic-acid model, magnocellular preoptic nucleus, medial septum, parvalbumin, neurophysiology

## Abstract

Temporal lobe epilepsy (TLE) is the most prevalent form of epilepsy, through the neuronal mechanisms of this syndrome remain elusive. In addition to the temporal lobe structures, it was found that the basal forebrain cholinergic cells are also involved in epileptogenesis. However, little is known about the involvement of the basal forebrain GABAergic neurons in epilepsy; despite this, they largely project to the temporal lobe and are crucial for the regulation of the hippocampal circuitry. In this study, we assessed epilepsy-induced changes in parvalbumin (PARV) immunoreactive neurons of the medial septum (MS) and of the magnocellular preoptic nucleus (MCPO) using the kainic acid (KA) model in rats. In addition, we estimated the respective changes in the cholinergic varicosities in the MS, where we observed a significant reduction in the PARV cell number (12849 ± 2715 vs. 9372 ± 1336, *p* = .029) and density (16.2 ± 2.62 vs. 10.5 ± 1.00 per .001 mm^3^, *p* =.001), and an increase in the density of cholinergic varicosities (47.9 ± 11.1 vs. 69.4 ± 17.8 per 30,000 µm^2^, *p* =.036) in KA-treated animals. In the MCPO, these animals showed a significant increase in somatic volume (827.9 ± 235.2 µm^3^ vs. 469.9 ± 79.6 µm^3^, *p* = .012) and total cell number (2268.6 ± 707.1 vs. 1362.4 ± 262.0, *p* =.028). These results show that the basal forebrain GABAergic cell populations undergo numerical and morphological changes in epileptic animals, which may contribute to an increased vulnerability of brain circuits to epilepsy and epilepsy-related functional impairments.

## 1. Introduction

Temporal lobe epilepsy (TLE) is a neurological disease affecting nearly 65 million people worldwide [1],[2]. It is characterized by recurrent unprovoked seizures associated with a profound reorganization of brain circuitry at both the cellular and molecular levels. This reorganization can lead to cognitive, psychological, neurobiological, and social consequences [2],[3].

The basal forebrain (BF) has been intensively studied since it has a key role in the processes of cortical activation, sleep homeostasis, attention, learning, and memory, which are compromised in TLE patients [3]–[7]. It has been suggested that the cholinergic system has a major contribution to TLE-related neurological alterations [8], with little focus on the other cell populations, namely GABAergic neurons.

The medial septum (MS) has been extensively studied in the context of TLE because it is the predominant source of cholinergic inputs to the hippocampal formation (HF), where it regulates high-frequency oscillations which can originate epileptic seizures [9],[10]. For example, we have previously reported that induction of epilepsy in rats triggers hypertrophic alterations in the MS cholinergic neurons and rearrangement of their terminals in the dentate gyrus [11]. Despite knowing that septal GABAergic cells are equally important for the regulation of hippocampal circuits, particularly by synchronizing local GABAergic interneurons in faster frequency domains, little is known of what happens to this specific population of cells within the epileptic brain [9],[10],[12].

In addition to MS, the magnocellular preoptic nucleus (MCPO), which also belongs to the BF, contains a great number of cholinergic and GABAergic cells [13]. It receives cholinergic inputs from the pedunculopontine (PPN) and laterodorsal tegmental (LDT) nuclei and serotonergic inputs from the dorsal raphe (DR) [14],[15]. Although the function of this nucleus has not yet been determined, it is believed that it is involved in the processes of memory formation and decision-making, since it projects to the medial prefrontal cortex, the entorhinal cortex, and limbic cortical areas [14]. Additionally, whether MCPO is affected by epilepsy is unknown. We previously found that unlike those located in the MS, cholinergic neurons located in the MCPO do not undergo hypertrophic alterations in epileptic rats [16], suggesting a low probability for its involvement in epileptogenesis.

The kainic acid (KA)-induced animal model is one of the best-characterized models of TLE. In fact, of all the existing TLE animal models, it is the one that best mimics the histopathologic findings observed in human patients [17],[18]. Using this model, we previously found morphological alterations in the PPN, LDT, and MS cholinergic neurons [11],[19], as well as in the serotonergic neurons of the DR nuclei [20]. In the present study, we aimed to evaluate the effects of epilepsy on GABAergic neurons located in the MS and MCPO. To achieve this goal, we stained brain sections against a Ca^2+^-binding protein parvalbumin (PARV), known to be co-expressed by the BF GABAergic projection neurons [21],[22]. Based on prior findings that KA-induced epilepsy in rats produces distinct effects on cholinergic cells located in the MS and MCPO, we hypothesized that changes in GABAergic cells, if present, can also be different in these two nuclei.

## 2. Methods

### 2.1. Ethical Statement

Animal handling and care were carried out in accordance with the Directive 2010/63/EU of the European Parliament and of the Council of September 22, 2010, on the protection of animals used for scientific purposes, as well as the “Principles of Laboratory Animal Care” (NIH publication No. 86-23, updated 1985). The General Veterinary Direction and the Ethics Committee of the Faculty of Medicine of the University of Porto have approved the experimental protocol for the FCT application grant PTDC/SAU-NSC/115506/2009 as of March 3, 2012. We ensured the use of the minimum number of animals and warranted their welfare.

### 2.2. Animals and Treatment

A total of 14 male Wistar rats were used in these experiments. At ten weeks of age, they were randomly divided into either the KA-treated group (9.5 mg/kg; n = 8) or the control group (n = 6), receiving equivalent volumes of KA and saline through intraperitoneal injections, respectively. We used a single high-dose KA treatment protocol because Wistar rats housed in our animal facilities displayed a reduced sensitivity to the low-dose repeated treatment protocol frequently used in other groups [23]. The animals were placed in individual boxes for behavioral observation for six hours. Motor seizures were assessed using the modified Racine scale for generalized motor seizures of focal limbic origin. *Status epilepticus* (SE) was defined as continuous seizures, which were scored as Racine's stage 3–5, with no recovery of “baseline” behavior (such as grooming, sniffing, and exploratory activity). Only rats that had SE for at least 3 h and showed at least one stage 5 seizure were included in further analyses. No seizure-like behavior was observed in the control group. Post-SE care consisted of evaluating the animal's general state twice daily and giving moisturized chow and subcutaneous saline injections. Two weeks after the injections with either KA or saline, the rats were monitored for the appearance of spontaneous motor seizures during at least two 2 h intervals per day by an investigator blinded to the treatment groups.

### 2.3. Tissue Preparation

Approximately six months after KA-injection, the animals were deeply sedated with pentobarbital (90 mg/kg) and transcardially perfused with 150 mL of 0.1 M phosphate-buffered saline (PBS; pH 7.4) for vascular washing, followed by 250 mL of a 4% paraformaldehyde fixative solution. The brains were removed and immersed for 2 hours in a fixative solution and afterward in a 10% sucrose solution for 36 hours at 4 °C. A vibratome was used to section the brains in the coronal plane at 60 µm to obtain sections containing the BF nuclei. Sections were kept in cryoprotectant (30% sucrose, 30% ethylene glycol, 0,25 mM polyvinylpyrrolidone in PBS) at −20 °C until histological processing.

### 2.4. Immunohistochemistry

From each brain, every third section containing the MCPO region and every sixth section containing the MS region were systematically sampled and immunostained against PARV. Additionally, MS sections were immunostained against the vesicular acetylcholine transporter (VAChT).

The brain sections were rinsed three times in PBS for a total of 30 minutes. Then, the endogenous peroxidases were removed by immersing the sections in a 1% H_2_O_2_ solution for 15 minutes. Next, the sections were washed six times in PBS for a total of 30 minutes and blocked for nonspecific staining through immersion into a solution containing 10% normal horse serum (NHS) + 0.5% Triton-X 100 in PBS for 2 h. Afterward, the sections were incubated with a primary anti-PARV antibody (Merck Millipore MAB1572; 1:5000 dilution) dissolved in PBS (1% NHS + 0,5% Triton-X) for 48 h at 4 °C. Then, sections were washed three times in PBS containing 2% NHS for a total of 30 minutes and subsequently incubated with a biotinylated secondary antibody anti-mouse (Vector Laboratories BA2000, 1:200 dilution) for 1 h. Next, the sections were washed three times for a total of 30 minutes and incubated with avidin-biotin-peroxidase complex (Vector Laboratories, Vectastain Elite ABC Kit) to perform the peroxidase reaction with 3,3′-diaminobenzidine (1 mg/mL) and H_2_0_2_ (0,08% in PBS). The sections were washed two times in PBS for a total of 20 minutes. After the staining procedures, sections were mounted on gelatin-coated slides, air-dried, dehydrated in a series of ethanol solutions (70%, 90%, 95%, and 100%), and coverslipped with Histomount (National Diagnosis, Atlanta, GA, USA).

For VAChT immunostaining, the aforementioned procedure was performed using a primary anti-VAChT antibody (Merck Millipore ABN100; 1:1000 dilution) and biotinylated secondary anti-goat antibody (Vector Laboratories BA-9500, 1:200 dilution).

### 2.5. Estimation of the Somatic Volume of PARV-Immunoreactive Neurons

The PARV-stained sections containing the MS and/or the MCPO regions were visualized using an Olympus BX-53 microscope equipped with a computer-controlled motorized stage system (MBF Bioscience, Williston, USA). The boundaries of the MS and MCPO nuclei were consistently defined using the previously described cytoarchitectonic criteria [24], and the rat brain atlas of Paxinos and Watson [25]. The optical fractionator probe of the Stereo Investigator software was used to sample the PARV-immunoreactive (PARV-IR) cells to measure their somatic volumes. The measurements were carried out using the nucleator probe for isotropic systematically random sampled sections, and the nucleolus of each cell was used as a central point. The coefficients of error of the individual estimate were inferior to 0.04 in all animals.

### 2.6. Estimation of the Total Number and cell density of PARV-Immunoreactive Neurons

A Carl Zeiss Axio Imager Z2 microscope equipped with a color digital camera and a computer-controlled motorized stage system (MBF Bioscience, Williston, USA) was used to view the PARV-stained sections. The boundaries of the MS and MCPO were consistently determined using the rat brain atlas of Paxinos and Watson [25], as well as the previously known cytoarchitectonic criteria [24]. The MCPO analysis was performed on one side of the brain, and randomly chosen for each animal. The total number of neurons was estimated using the optical fractionator probe of the Stereo Investigator software (MBF Bioscience). A 5x objective lens was used to outline the areas of interest, and a 63x oil-immersion lens was used to count the neurons. Using the raster pattern method, counting frames were systematically sampled from a random beginning position inside the region of interest. Tissue thickness was estimated at each counting frame, and guard zones of 1 µm were implemented. The nucleus of the neurons was used as the counting unit. Systematically sampled sections containing the MS and MCPO regions were used, yielding an average of 6 and 4 sections per rat, respectively. The error coefficients in the individual estimations, which ranged between 0.07 and 0.12 for MS sections and between 0.08 and 0.12 for MCPO sections, were determined using Gundersen et al. [24]. To estimate the density of the PARV-IR neurons, we divided the total number in each animal by the respective sampled volume (dissector volume, in mm^3^, multiplied by the number of sampling sites).

### 2.7. Density of VAChT-Immunoreactive Varicosities in the MS

From each brain, four consecutive VAChT-stained slices containing the MS were sampled. Using the rat brain atlas of Paxinos and Watson [25], the sections were level-matched across all animals included in the analysis. The sections were visualized using an Axio Scope.A1 microscope equipped with a Leica EC3 color digital camera. In each section, two photomicrographs were taken with a 100× objective lens. Using the Fiji image-processing software (http://rsb.info.nih.gov/ij/), two non-overlapping counting frames of 1,274 µm^2^ each were applied to each image, and the number and cross-sectional area of all varicosities that fell inside each frame were calculated. The varicosity densities obtained were standardized to an arbitrary area of 30,000 µm^2^ and averaged over all sections per animal. Cross-sectional areas ranging from .11 to .21 µm^2^ were included.

### 2.8. Statistical Analysis

All the data obtained in this study satisfied the assumption of normality (Shapiro-Wilk's W test; *p* > .05). A Multivariate Analysis of Variance (MANOVA) test was used for comparing the control and KA groups regarding the PARV cell number, PARV cell density, and VAChT-stained varicosities in the MS. For the MCPO, a MANOVA was also used to compare the PARV-stained cell volume, number, and density between groups. Subsequent ANOVA tests were applied for significant MANOVA results. Differences were considered significant at the *p* < .05 level.

## 3. Results

### 3.1. Seizures and Mortality

Following KA injection, five out of the eight rats in the KA group exhibited a typical pattern of SE and reached at least one stage 5 seizure. Two rats treated with KA died in the first-week post-KA injection, despite all efforts to reduce the mortality rate. One of the KA-treated rats did not exhibit behavioral seizures (seizures scoring stages 1–2 on the Racine scale). As a result, the final size of the KA group was five animals (n = 5). Seizures were not observed in rats that received saline injections (the control group).

### 3.2. Total number and density of PARV-Immunoreactive neurons and VAChT-Immunoreactive fiber varicosities in MS

**Figure 1.**
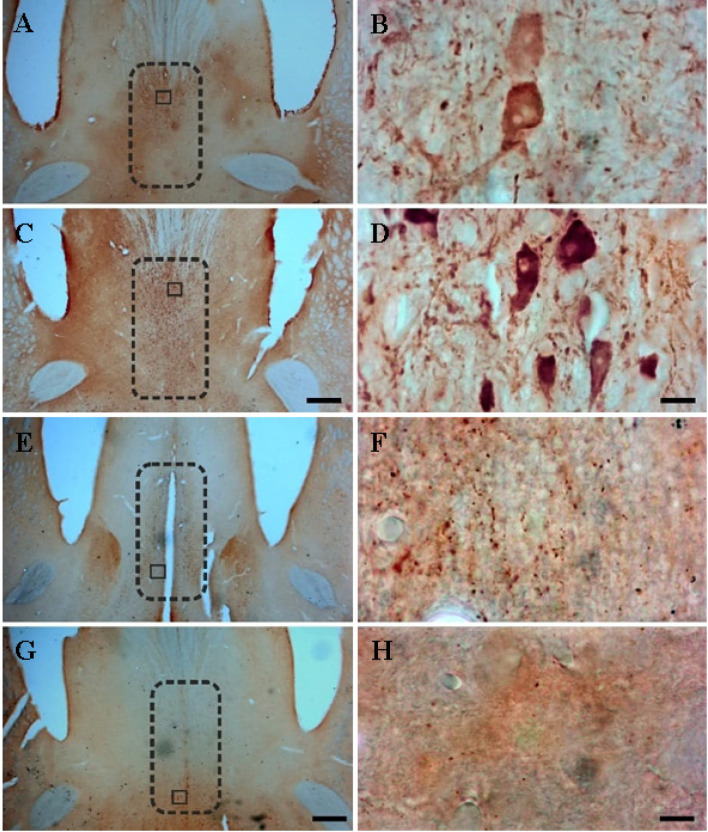
Representative photomicrographs of the medial septum nucleus of a kainic acid-treated rat **(A, B, E, F))** and of a control rat **(C, D, G, H)**. The sections were immunostained for PARV **(A-D)** or for VAChT **(E-H)**. The approximate boundaries of the medial septum nucleus are delineated with a dotted line **(A, C, E, G)**. Smaller insets seen in **(A)**, **(C)**, **(E)**, and **(G)** are shown as higher-power images in **(B)**, **(D)**, **(F)**, and **(H)**, respectively. Note the decreased number and density of PARV-positive neurons from the kainic-treated rat **(A, B)** versus the control rat **(C, D)**. In contrast, the density of VAChT-positive varicosities appears to be higher in the KA-treated rat **(E, F)** when compared to the control rat **(G, H)**. Scale bar = 500 µm **(A, C, E, F)**, and 10 µm **(B, D, F, H)**.

**Figure 2.**
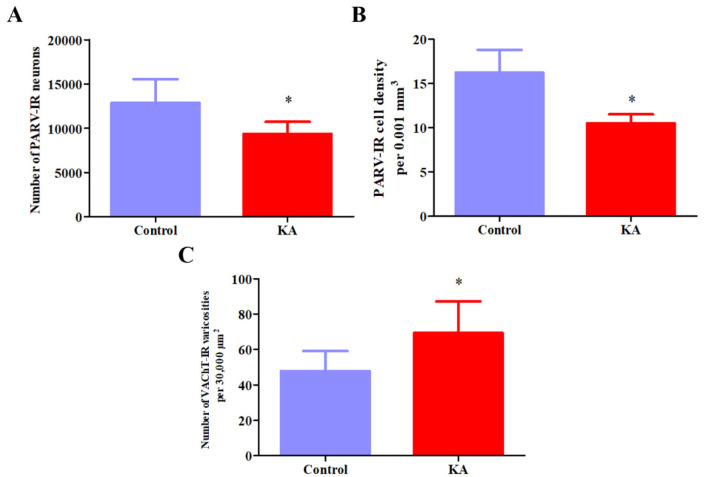
The effects of treatment with kainic acid on the total number and density of the MS PARV-stained neurons and on the density of the MS VAChT-stained fiber varicosities as a function of their cross-sectional area. **(A)** The medial septum of KA-treated rats presented a decrease of PARV-stained cells of approximately 27% when compared to controls. **(B)** Similarly, the density of PARV-stained neurons was decreased by approximately 35% in KA-treated rats when compared to controls. **(C)** In contrast, an increase in the density of the VAChT-stained varicosities of approximately 45% was observed in KA-treated animals when compared to controls. *p < .05.

Figure 1 shows representative photomicrographs of PARV- and VAChT-stained brain slices containing the MS. In the KA group, a reduction in PARV cell number (12849 ± 2715 vs. 9372 ± 1336, Fig. 2A) and density was observed (16.2 ± 2.62 vs. 10.5 ± 1.00 per .001 mm^3^, Fig. 2B), accompanied by an increase in density of VAChT varicosities (47.9 ± 11.1 vs. 69.4 ± 17.8 per 30,000 µm^2^, Fig. 2C). A MANOVA showed a significant group effect on the three dependent variables (Wilks' lambda = .19, F(3,7) = 9.71, *p* = .007, η_p_^2^ = .81). Significant changes in PARV cell number (F(1,9) = 6.74, *p* = .029, η_p_^2^ = .43) and density (F(1,9) = 21.2, *p* = .001, η_p_^2^ = .70), and density of VAChT-stained varicosities (F(1,9) = 6.03, *p* = .036, η_p_^2^ = .40) were found between groups using an ANOVA.

### 3.3. Somatic volume, total number, and density of PARV-immunoreactive neurons in MCPO

**Figure 3.**
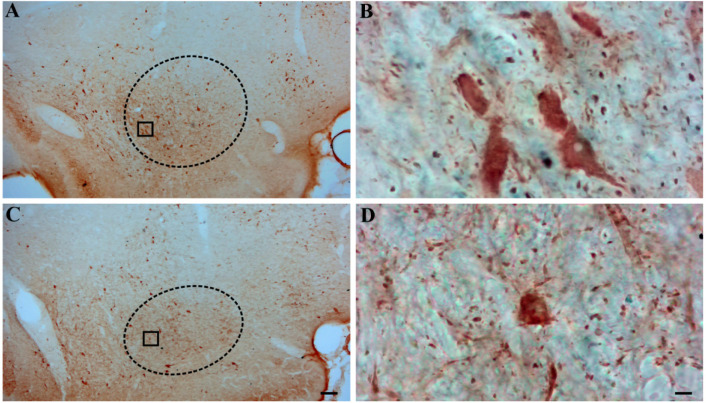
Photomicrographs of representative PARV-stained coronal sections containing the magnocellular preoptic nucleus taken from a kainic acid-treated rat with confirmed spontaneous seizures (A, B) (top panels) and from a control rat (C, D) (lower panels). The approximate boundaries of the magnocellular preoptic nucleus are delineated with a dotted line. Insets in **(A)** and **(C)** are shown as higher-power images in **(B)** and **(D)**, respectively. The number of PARV-positive cells and their mean somatic volume appears to be higher in the KA-treated rat **(A, B)** when compared to the control rat **(C, D)**. However, the density of cells stained for PARV appears to be similar in the two groups. Scale bar = 200 µm **(A, C)** and 10 µm **(B, D)**.

**Figure 4.**
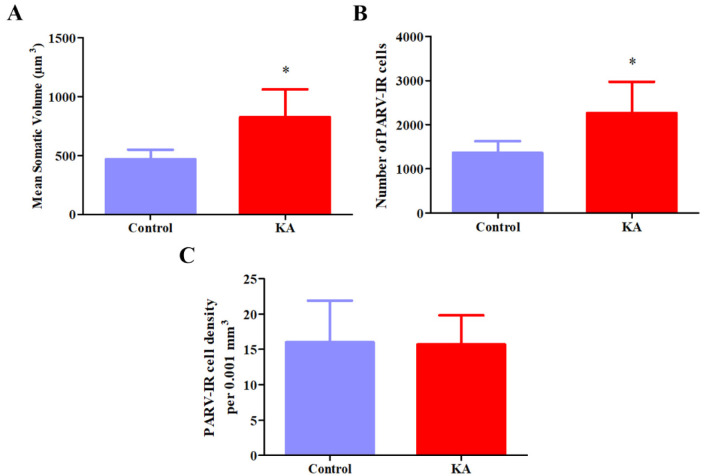
The effects of treatment with kainic acid on the mean somatic volume, total number, and density of PARV-stained neurons in the magnocellular preoptic nucleus. **(A)** The mean somatic volume of PARV-stained neurons presented an increase of approximately 76% in KA-treated rats when compared to controls. **(B)** Similarly, the total number of PARV-stained cells was increased by approximately 66,5% in KA-treated rats when compared to controls. **(C)** In contrast, no significant differences in the density of PARV-stained cells were found between the groups. *p < .05.

Representative photomicrographs of PARV-stained brain slices containing the MCPO are shown in Figure 3. The stereological analyses were not performed in one animal from the control group due to an insufficient number of sections containing the MCPO. A MANOVA showed a significant group effect on the volume, total number, and density of the PARV-IR neurons in this nucleus (Wilks' lambda = .097, F(3,6) = 18.6, *p* = .002, ηp2 = .91). An ANOVA revealed that the KA group had a significant increase in somatic volume (827.9 ± 235.2 µm^3^ vs. 469.9 ± 79.6 µm^3^, F(1,8) = 10.4, *p* = .012, η_p_^2^ = .57) and total cell number (2268.6 ± 707.1 vs. 1362.4 ± 262.0, F(1,8) = 7.22, *p* = .028, η_p_^2^ = .47), but not in cell density (15.7 ± 4.11 vs. 16.0 ± 5.88 per .001 mm^3^, F(1,8) = .013, *p* = .91, η_p_^2^ = .002). The stereological estimates of the mean somatic volumes, total number, and density of these cells are illustrated in Figure 4A–C.

## 4. Discussion and conclusion

An imbalance between the excitatory and the inhibitory components of the brain circuitry is recognized as the underlying mechanism for TLE. The cholinergic BF cells have been widely studied, since they provide most of the cholinergic input to the HF and are crucial in regulating hippocampal circuits. However, relatively few studies have assessed the role of hippocampal-projecting GABAergic cells which are just as, if not more, important for the regulation of neuronal excitability. The key finding of this study is that BF GABAergic projection neurons suffer significant morphological and numerical changes in the rat epileptic brain. Furthermore, these changes qualitatively differ between the two BF subdivisions, MS and MCPO, likely due to the distinct involvement of these nuclei in epileptogenesis.

We previously reported that KA-induced SE produces a significant rearrangement in the septohippocampal and brainstem-thalamic cholinergic systems. Additionally, our group demonstrated a significant reorganization of ascending serotoninergic pathways in this model. In particular, numerical and hypertrophic changes, as well as considerable reorganization of efferent terminals, were observed in the MS, PPN and LDT, and DR, respectively [11],[19],[20]. In the present study, we extend this evidence to the BF GABAergic cells. Consistent with prior reports [27], we found that epilepsy leads to a loss of GABAergic cells in the MS (PARV-coexpressing subpopulation). Though one of the important roles of these cells is to inhibit hippocampal local circuit interneurons [9],[10],[12], their loss may not necessarily lead to an enhanced inhibition of hippocampal circuits for at least two reasons. First, in the very same conditions, MS cholinergic neurons show hypertrophic alterations accompanied by the reorganization of their terminals in the HF [19] and the MS itself (present study). Second, because seizures kill many local circuit GABAergic cells in the HF, fiber terminals of GABAergic cells, whose perikarya are in the MS, can be redistributed to the hippocampal principal (glutamatergic) cells. Thus, their loss can actually disinhibit the principal hippocampal neurons. This interpretation is consistent with prior studies, which revealed that MS GABAergic neurons are functionally altered by epileptogenesis [28]. Indeed, close-loop stimulation of these neurons has been shown to reduce seizure duration or even end epileptic seizures [29],[30].

Because we previously detected no epilepsy-related alterations in the cholinergic neurons of the MCPO [16], unlike that of the MS [19], we expected no alterations in MCPO GABAergic cells. Contrary to our expectations, the total number of the PARV-IR cells was significantly increased in the MCPO of epileptic rats. The GABAergic downregulation in the MS and upregulation in the MCPO suggest that the chronic state of epilepsy is associated with global rearrangements in the basal forebrain neurochemistry and neuroanatomy. It can be speculated that a loss of GABAergic cells and upregulation of cholinergic cells in the MS and diagonal band of Broca lead to increased neuronal excitability in epileptogenic brain regions, such as the hippocampus and amygdala; alternatively, upregulation of GABAergic transmission in the MCPO, which sends only a small portion of afferents to the hippocampus and amygdala, may result in neuronal inhibition in other brain regions where this nucleus preferentially projects, namely in the prefrontal cortex and olfactory brain regions. Indeed, MCPO has been linked to the processes of olfactory discrimination and memory [31]–[33], as well as of cortical arousal and wakefulness maintenance [34]. Furthermore, given its strategic location (i.e., receiving afferents from the DR and PPN and projecting to the olfactory and limbic cortical areas), it may act as a link between the cholinergic brainstem-thalamic, ascending serotoninergic, and septohippocampal pathways [14],[15]. Thus, it may contribute to the heterogeneous presentation of seizures in TLE and to the wide spectrum of comorbidities that might involve multiple brain regions with different functions, such as memory and decision-making. However, further research is needed to clarify the role of MCPO in this context and to better understand the biological implications of the increased GABAergic drive of this nucleus upon its target neurons.

Both MS and MCPO contain neurons of different chemotypes, such as cholinergic, GABAergic, and glutamatergic neurons, and many MS GABAergic neurons use acetylcholine as a co-neurotransmitter [35]–[37]. Furthermore, it is important to mention that the BF GABAergic populations can be further divided into different subpopulations based on the expression of calcium-binding proteins, namely PARV, calbindin-D28k, and calretinin [38]. A growing body of evidence suggests that the dual-transmitter phenotype may vary through different conditions, both in healthy and pathological circumstances [39]. Hence, future research should look at whether the induction of epilepsy affects other neuronal populations of the MS and MCPO, in addition to those immunoreactive to PARV and VAChT, and whether it can lead to phenotypic changes in dual-transmitter cells.

In animal models of epilepsy, MS cholinergic neurons have been shown to suffer hypertrophic changes [11],[40]. In this study, we provide evidence that the soma of GABAergic cells, namely those located in the MCPO, can also be considerably enlarged in this condition. Interestingly, it has been proposed that perikaryal enlargement in epilepsy can reflect increased metabolic demands, such as neurite outgrowth [36]. Additionally, neuronal hypertrophy can be viewed as an adaptive response to brain damage caused by either an initial insult or excessive neuronal excitation during recurrent spontaneous seizures [41],[42]. Indeed, seizure activity has been demonstrated to activate the mammalian target of the rapamycin (mTOR) signaling pathway, which is involved in controlling cell size, growth, and proliferation [43]. Thus, future research should attempt to shed light on the molecular mechanisms underlying the plasticity of GABAergic and cholinergic neurons. Uncovering the biochemical pathways and physiological mechanisms that cause hypertrophic changes in the BF neurons may provide new molecular targets for the development of novel therapeutic approaches to drug-resistant types of epilepsy.

In summary, the present findings provide evidence that the BF GABAergic cell populations undergo a variety of numerical and morphological alterations in the brains of TLE animals that may contribute to the increased vulnerability of brain circuits to epilepsy and epilepsy-related functional impairments.
